# Epitope mapping by a Wnt-blocking antibody: evidence of the Wnt binding domain in heparan sulfate

**DOI:** 10.1038/srep26245

**Published:** 2016-05-17

**Authors:** Wei Gao, Yongmei Xu, Jian Liu, Mitchell Ho

**Affiliations:** 1 Laboratory of Molecular Biology, Center for Cancer Research, National Cancer Institute, National Institutes of Health, Bethesda, MD 20892, USA; 2 Key Laboratory of Human Functional Genomics of Jiangsu Province, Department of Cell Biology, School of Basic Medical Science, Nanjing Medical University, Nanjing, Jiangsu, 210029, China; 3 Division of Chemical Biology and Medicinal Chemistry, Eshelman School of Pharmacy, University of North Carolina, Chapel Hill, NC 27599, USA

## Abstract

Heparan sulfate (HS) is a polysaccharide known to modulate many important biological processes, including Wnt signaling. However, the biochemical interaction between HS and Wnt molecules is not well characterized largely due to the lack of suitable methods. To determine the Wnt binding domain in HS, we used a Wnt signaling-inhibitory antibody (HS20) and a panel of synthetic HS oligosaccharides with distinct lengths and sulfation modifications. We found that the binding of HS20 to heparan sulfate required sulfation at both the C2 position (2-O-sulfation) and C6 position (6-O-sulfation). The oligosaccharides with the greatest competitive effect for HS20 binding were between six and eight saccharide residues in length. Additionally, a four residue-long oligosaccharide could also be recognized by HS20 if an additional 3-O-sulfation modification was present. Furthermore, similar oligosaccharides with 2-O, 6-O and 3-O-sulfations showed inhibition for Wnt activation. These results have revealed that HS20 and Wnt recognize a HS structure containing IdoA2S and GlcNS6S, and that the 3-O-sulfation in GlcNS6S3S significantly enhances the binding of both HS20 and Wnt. This study provides the evidence for identifying the Wnt binding domain in HS and suggests a therapeutic approach to target the interaction of Wnt and HS in cancer and other diseases.

Heparan sulfate proteoglycans (HSPGs) are involved in many biological processes, including early development^1^, tumor growth^2^,^3^,^4^ and viral infections^5^. They can interact with multiple types of extracellular and cell surface factors. HSPGs can function as co-receptors or as cell surface storage sites used to recruit these growth factors. They also facilitate receptor-ligand interactions by binding and localizing specific growth factors, which can increase their local biological effects^6^. HSPG contains both a core protein and heparan sulfate (HS) polysaccharide side chains. The regulatory roles displayed in these biological processes are mainly mediated by the HS chains^2^,^7^. HS chains are heterogeneous in both the length of their polysaccharide chains and in the sulfations that modify these chains. HS contains repeating disaccharides made of N-acetyl-glucosamine (GlcNAc) and glucuronic acid (GlcA). These repeating disaccharides are most frequently modified via sulfation at the 2-O and 6-O positions, with relatively infrequent modification at the 3-O position^8^. The position of these sulfation modifications are precisely regulated by enzymatic reactions that occur along the chain^9^.The functional domains are usually 3 to 6 disaccharides in length^10^ and serve as docking sites for factors such as fibroblast growth factor (FGF) and anti-thrombin^11^,^12^.

HS has an extremely heterogeneous structure due to the position of sulfation, the length of the sulfated domain and the spacing between fragments. In addition, post-synthesis events contribute to the diversity of HS structure. Enzymes such as sulfatases, which catalyze the hydrolysis of 6-O-sulfation from HS polysaccharides, and heparanases, which cleave the HS chains at different sites, further contribute to the dynamic structure of HS^11^. Therefore, it remains a challenge to distinguish among the many manifestations of HS and to determine their corresponding functions. Sulfatase and heparanase are widely used as research tools to define HS-related functions^13^,^14^,^15^,^16^,^17^. The HS and heparan being studied represent a small percentage of the possible structures since they are obtained from a few tissues originating from a limited number of species. There is a huge variety of HS that exists in the natural world, so a broader strategy is necessary. Although HS metabolic enzymes can be used to track changes in HS, these enzymatic treatments preferentially show the outcome of changes across a population instead of a single type of HS oligosaccharide.

Wnt signaling has been shown to play an essential role in early development^18^,^19^ and tumorigenesis^20^. HSPGs can modulate Wnt activation as co-receptors^21^. Glypicans and sydecans are the two major types of HSPGs. Both of these chains can bind Wnt and Frizzled, and therefore potentially enhance Wnt activation at the cell surface^22^,^23^. Many studies show that the HS chains of HSPGs are crucial for Wnt binding^24^,^25^. Additionally, Wnt signaling can be modified by treating the HS with metabolic enzymes such as glycosylation transferases^26^ and sulfatases^27^,^28^. However, the biochemical interaction of HS and Wnt remains unclear.

Glypican-3 (GPC3) is a cell surface heparan sulfate proteoglycan that is highly expressed in hepatocellular carcinoma (HCC)^29^,^30^,^31^. It has been shown that GPC3 interacts with Wnt3a and promotes HCC cell proliferation^32^,^33^,^34^,^35^. Using phage display technology, we isolated a high-affinity human monoclonal antibody (HS20) that recognizes the HS chains of GPC3. We found that HS20 disturbed the interaction between GPC3 and Wnt3a, blocked Wnt activation, inhibited Wnt3a-induced HCC cell proliferation and showed anti-tumor activity in mice^32^. Our observations have indicated the therapeutic value of HS20 because the antibody functions as a novel Wnt-blocking molecule by binding tumor-specific GPC3 instead of conventional Wnt or Frizzled molecules. Interestingly, several other glypicans, including glypican-1 (GPC1) and glypican-5 (GPC5), can also be recognized by HS20^36^, indicating that the highly conserved HS epitope serves as the binding site for the antibody. Currently, the HS-Wnt interaction remains poorly characterized largely due to the lack of suitable methods and materials. In the present study, we used the HS20 antibody and a panel of HS oligosaccharides with distinct properties as research tools to dissect the HS structure required for Wnt binding. We compared HS20-binding saccharide sequences to the HS structure preferred for Wnt binding. We found that (1) HS20 specifically targeted HS, not chondroitin sulfate (CS); (2) polysaccharides recognized by HS20 required both 2-O and 6-O sulfations with a length between six and twelve saccharide residues; and (3) four saccharide residues with an extra 3-O-sulfation were sufficient for antibody binding. Moreover, HS20 binding and Wnt binding shared the similar HS oligosaccharide structure. This study provided the initial evidence for the Wnt binding domain on HS and supports a rationale to block the Wnt binding site on HS for therapeutics development.

## Results

### The HS20 human monoclonal antibody is specific for HS

**Figure 1:**
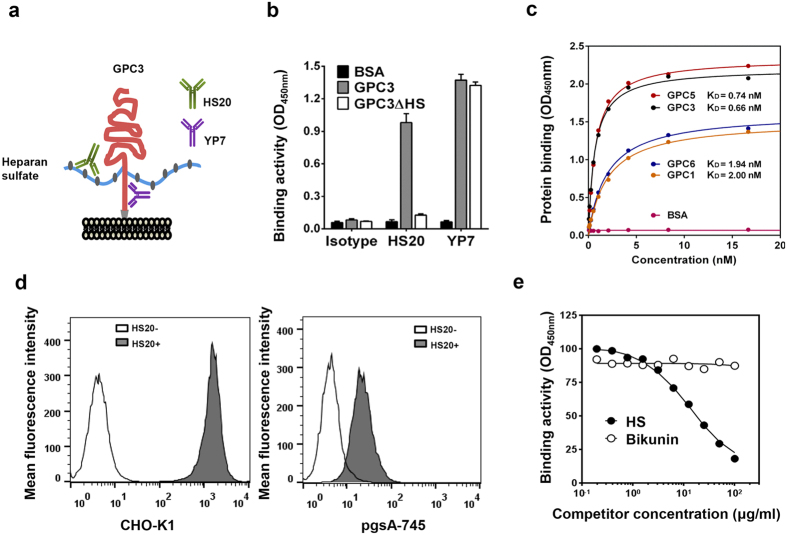
The binding properties of HS20 monoclonal antibody. (**a**) The schematic structure of GPC3, the human monoclonal antibody HS20 recognizing the heparan sulfate chains on GPC3 and the mouse monoclonal antibody YP7 recognizing a C-terminal region of the GPC3 core protein. (**b**) HS20 did not bind the mutant GPC3 without heparan sulfate chains (GPC3ΔHS). ELISA results showed the binding properties of HS20 and YP7 on wild type GPC3 and GPC3ΔHS. BSA was used as the negative antigen control. Values represent mean ± SD, t-test. (**c**) The binding property of HS20 to various glypicans. ELISA results show the binding affinities of HS20 for GPC1, GPC3, GPC5 and GPC6. The X-axis represents the concentration of HS20, while the Y axis indicates protein binding as determined by the OD_450 nm_. (**d**) HS20 lost binding capacity on the cells without heparan sulfate. Flow results showed the binding of HS20 for CHO-K1 cells and pgsA-745 cells, which lack the ability to produce HS and CS. (**e**) HS but not CS inhibited HS20 binding for GPC3. A competitive ELISA where HS20 (5 μg/ml) was pre-incubated with increasing concentrations of HS or bikunin (CS) for 30 minutes, and then added to an ELISA plate to detect the binding to GPC3.

In a previous study, we generated two anti-GPC3 monoclonal antibodies, HS20 and YP7. GPC3 is a cell surface proteoglycan highly expressed on HCC cells (Fig. 1a). We found that HS20 failed to bind the mutant GPC3 lacking HS chains, indicating that HS20 recognizes the HS chains on GPC3 and not the core protein. The control YP7 antibody bound the core protein of GPC3 because it was capable of binding both wild type GPC3 and the mutant GPC3 lacking HS chains (Fig. 1b). We found that HS20 also recognized other glypicans (GPC1, GPC5 and GPC6) (Fig. 1c). This indicates that the binding site for HS20 is a highly conserved HS structure that is shared between glypicans. To analyze the binding property of HS20, we tested its binding on pgsA-745 cells. This cell line is a mutant CHO-K1 cell line that lacks xylosyltransferase^37^,^38^. As a result, it is incapable of producing glycosaminoglycans, which include HS and chondroitin sulfate (CS). We found that the binding of HS20 on pgsA-745 cells was dramatically reduced (Fig. 1d), indicating that HS20 cannot recognize the cells without HS or CS modifications. To examine whether HS20 is capable of binding to CS chains, we performed a competitive ELISA using HS and bikunin^39^, a proteoglycan with only CS. As shown in Fig. 1e, HS inhibited the binding of HS20 on GPC3, but bikunin had no effect. Taken together, our results showed that HS20 specifically recognizes HS, but not CS. The different effect observed for bikunin and HS supports the conclusion that the binding between HS and HS20 is not due to a purely negative charge effect, since both compounds have similar numbers of negative charges. Therefore, this data also indicates that HS20 binding is not charge-dependent.

### The HS20 binding site on HS requires both 2-O-sulfation and 6-O-sulfation

**Figure 2:**
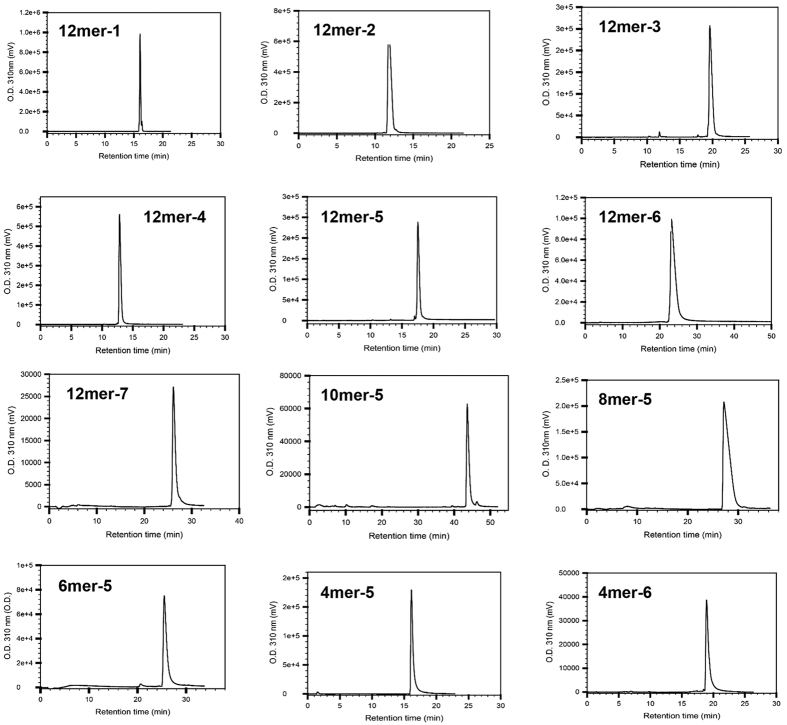
HPLC chromatograms of synthesized oligosaccharides. Both DEAE-HPLC and PAMN-HPLC methods were used to analyze the purity of each individual oligosaccharide. Overall, the purity for each compound was determined to be >90%.

**Figure 3:**
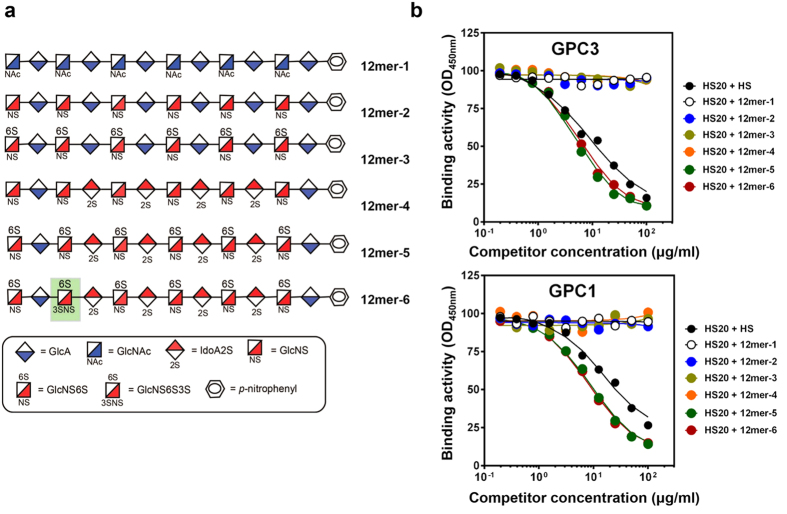
The binding of HS20 required both 2-O-sulfation and 6-O-sulfation. (**a**) Structures of the synthetic oligosaccharides (12mers) with different sulfations. (**b**) Both 2-O-sulfation and 6-O-sulfation were required for HS20 binding in a competitive ELISA. Indicated oligosaccharides were pre-incubated with the HS20 antibody for 30 minutes, and then the mixture was added into an ELISA plate to detect the binding of GPC3 or GPC1. HS was used as a positive control inhibitor.

To further investigate what structural motif of HS is recognized by HS20, we synthesized HS oligosaccharides for the present study using a chemoenzymatic method^40^,^41^. The purity of each oligosaccharide was greater than 90% as determined by high resolution DEAE-HPLC (Fig. 2), and the structures were confirmed by electrospray ionization mass spectrometry (ESI-MS) (Table 1). As shown in Fig. 3a, dodecsaccharides12mer-1 and 12mer-2 lacked O-sulfated modification; 12mer-3 had 6-O-sulfation (GlcNS6S), 12mer-4 had 2-O-sulfation (IdoA2S), and 12mer-5 had both 6-O-sulfation and 2-O-sulfation. The competitive ELISA results showed that only oligosaccharides containing both GlcNS6S and IdoA2S (12mer-5) significantly inhibited the binding of HS20 on GPC3 (Fig. 3b). When we added an extra 3-O-sulfation on GlcNS6S, the oligosaccharide (12mer-6) exhibited similar inhibition of HS20 binding compared to 12mer-5. Furthermore, these oligosaccharides showed similar inhibition patterns when tested against GPC1. This data suggests that the binding of HS20 to HS requires both 2-O-sulfation and 6-O-sulfation.

**Figure 4:**
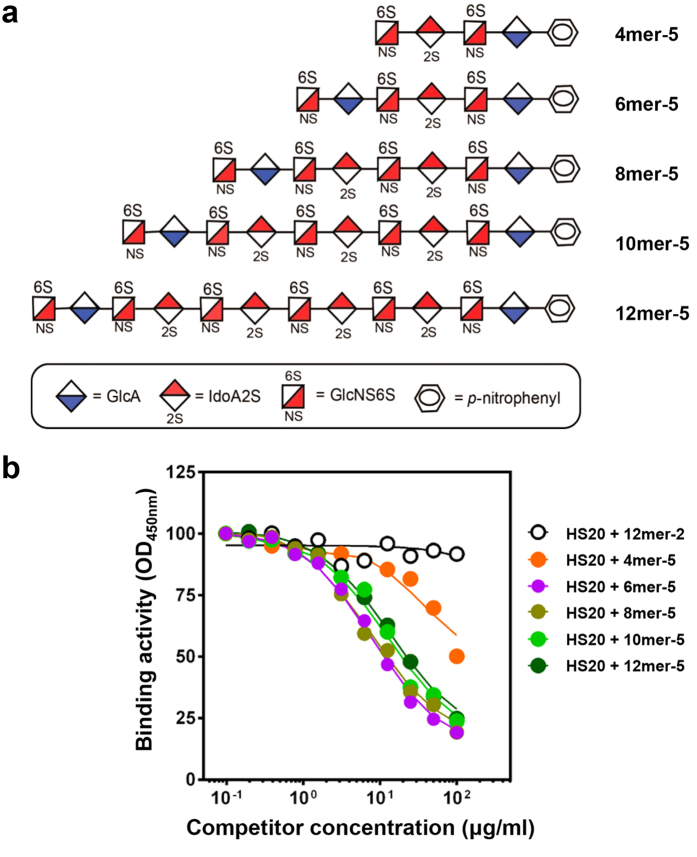
The minimal HS binding motif of HS20 was a 6mer with both 2-O-sulfation and 6-O-sulfation. (**a**) Structures of the synthetic oligosaccharides (4mer to 12mer) with both 2-O and 6-O-sulfation. (**b**) The 2-O and 6-O sulfated oligosaccharides with lengths longer than 4mer inhibited HS20 binding. Competitive ELISA was performed by pre-incubating oligosaccharides with HS20 for 30 minutes, and then adding the mixture into an ELISA plate to detect the binding to GPC3.

To determine whether the length of the oligosaccharides would affect HS20 binding, we used a group of oligosaccharides with varying lengths (Fig. 4a). Interestingly, 6mer-5 and 8mer-5 showed the most dramatic inhibition of HS20 binding; 10mer-5 and 12mer-5 exhibited competitive effects that were slightly less than those of 6mer and 8mer. The oligosaccharide 4mer-5 only partially inhibited HS20 binding even at the highest concentrations tested (Fig. 4b). Our data suggests that the shortest oligosaccharide for the HS20 binding site consists of 6 saccharide residues.

### 3-O-sulfation on 4mer enhanced HS20 binding

**Figure 5:**
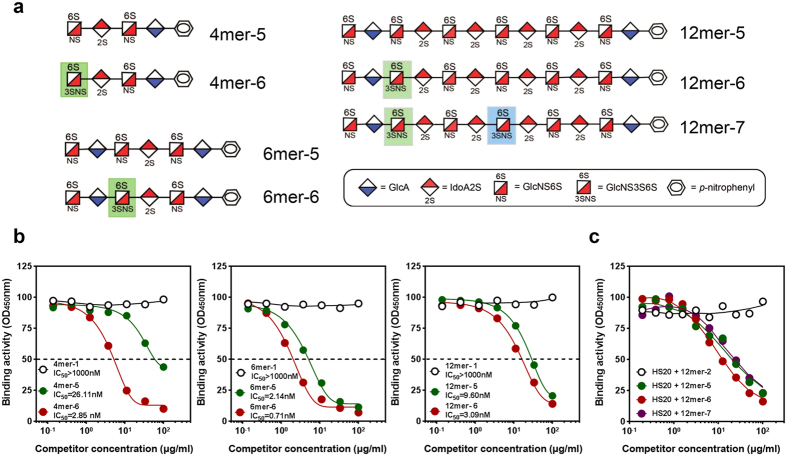
The addition of a 3-O-sulfation to oligosaccharides further inhibits HS20 binding. (**a**) Structures of the synthetic oligosaccharides (4mer to 12mer) with both 2-O and 6-O-sulfations (mer-5), with an extra 3-O-sulfation (mer-6), and with two extra 3-O-sulfations (mer-7). (**b**) The oligosaccharides with an extra 3-O-sulfation showed more inhibition of HS20 binding. Competitive ELISAs were performed on various 4mer, 6mer and 12mer oligosaccharides. Dashed lines indicate the value of IC_50_. (**c**) A competitive ELISA with various 12mers that have differences in the sulfation position and number.

To investigate whether 3-O-sulfation was involved in HS20 binding, we compared the inhibitory effects of HS20 binding on 4mer, 6mer, and 12mer oligosaccharides with or without 3-O-sulfation (Fig. 5a). The oligosaccharides all contained 2-O-sulfation and 6-O-sulfation, with a few of the oligosaccharides containing 3-O-sulfations. Interestingly, those 3-O-sulfated oligosaccharides (4mer-6, 6mer-6 and 12mer-6) exhibited significantly stronger inhibition of HS20 binding. The greatest effect of 3-O-sulfation was observed for the 4mer, which showed a tenfold increase in inhibition. Interestingly, the impact of 3-O-sulfation is decreased for larger oligosaccharides; the increased binding of HS20 on 6mer and 12mer was only threefold (Fig. 5b). These results indicate that when 3-O-sulfations are present, HS20 can recognize oligosaccharides as short as a 4mer. The addition of a second 3-O-sulfation to the 12mer oligosaccharide (12mer-7) did not result in an increased inhibition (Fig. 5c). Therefore, the second 3-O-sulfation did not further enhance HS20 binding.

### The oligosaccharides recognized by HS20 are capable of binding Wnt

**Figure 6:**
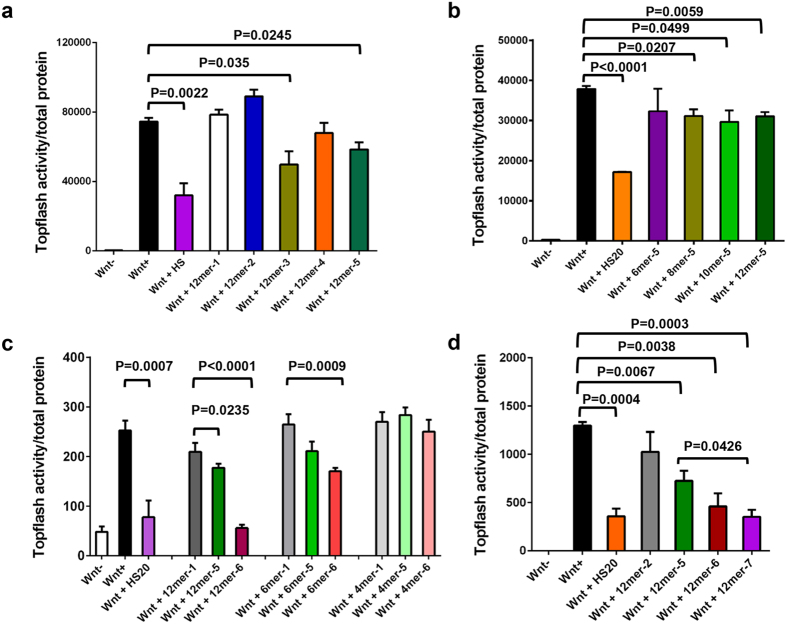
The effect of oligosaccharide variation on Wnt binding. (**a**) Wnt binding HS required 6-O-sulfation. Topflash assay: 50% Wnt3aCM was pre-incubated with HS or indicated oligosaccharides for 30 minutes and then added to HEK293Topflash cells. Luciferase activity was measured 6 hours later and normalized by total protein. HS was set up as a positive inhibitor. (**b**) 8mer, 10mer and 12mer polysaccharides with 2-O and 6-O sulfations inhibited Wnt activation. A Topflash assay was conducted as with 6mer, 8mer, 10mer, and 12mer oligosaccharides that contained 2-O and 6-O sulfations. The experiment followed the same procedure that was previously mentioned. (**c**) A 3-O sulfation on 2-O and 6-O sulfated oligosaccharides had enhanced Wnt inhibition. A Topflash assay was used to determine the effect of adding a 3-O sulfation on 2-O and 6-O sulfated oligosaccharides had enhanced Wnt inhibition to the various length oligosaccharides on Wnt signaling. The experiment followed the same procedure that was previously mentioned. (**d**) Two extra 3-O sulfations on 2-O and 6-O sulfated oligosaccharides showed further inhibition on Wnt activation. A Topflash assay was used to determine the effect of adding a second 3-O sulfation to a 12mer oligosaccharide. For all figures, values represent mean ± s.d.

Our previous study demonstrated that HS20 blocked the interaction of Wnt3a and GPC3 by targeting the HS chains on GPC3^32^. Thus, the oligosaccharides recognized by HS20 provide clues to the unknown Wnt binding site on HS. We used HEK293 Supertopflash, a HEK293 cell line expressing a Wnt reporter gene^42^, to examine Wnt activation in the presence of Wnt3a-conditioned medium and various HS oligosaccharides. As shown in Fig. 6a, 12mer-3 and 12mer-5 oligosaccharides inhibited Wnt activation, suggesting that 6-O-sulfation played an important role in Wnt binding. In addition to the sulfated positions, the length of polysaccharides also affects Wnt binding. The 2-O and 6-O sulfated 8mer-5, 10mer-5 and 12mer-5 (but not 6mer-5) oligosaccharides showed significant inhibitory effects on Wnt binding (Fig. 6b). To evaluate the effect of 3-O-sulfation on Wnt binding, we tested 3-O-sulfated oligosaccharides in the Wnt activation assay. The oligosaccharides with 3-O-sulfation (12mer-6 and 6mer-6, but not 4mer-6) showed enhanced inhibition upon Wnt activation (Fig. 6c). When the doubly 3-O-sulfated 12mer-7 was used, it also exhibited enhanced Wnt-blocking effects when compared to the 12mer-5 (Fig. 6d). However, the additional 3-O-sulfation in 12mer-7 did not significantly enhance Wnt inhibition as compared to 12mer-6.

**Figure 7:**
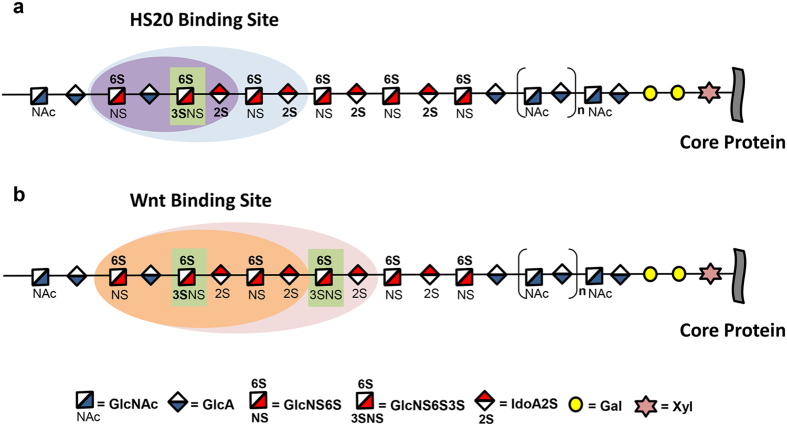
A comparison of Wnt and HS20 binding motifs on oligosaccharides. (**a**) Hypothetical schematic structures for HS20-binding HS. HS20-binding oligosaccharides require both 2-O and 6-O sulfations (bold). The site of 3-O-sulfation (bold) is shown by a green box. The minimal oligosaccharide recognized by HS20 is shown as a 6mer (light blue region) or as a 4mer with a 3-O-sulfation (purple region). (**b**) Hypothetical schematic structures for Wnt-binding HS. Wnt-recognized oligosaccharides require 6-O sulfation (bold). The sites of 3-O-sulfation are shown by green boxes. The minimal oligosaccharide recognized by Wnt is an 8mer (light pink region) or a 6mer with a 3-O-sulfation (orange region).

Taken together, our results showed that 6-O-sulfation and 2-O-sulfation were required for HS20 binding and that 6-O-sulfation was essential for Wnt binding. While 3-O-sulfation was not essential, it significantly enhanced binding activity for both HS20 and Wnt. Furthermore, Wnt preferred longer HS binding units (>6mer) as compared to HS20 (>4mer) (Fig. 7). This finding also validated our hypothesis that the HS motif recognized by HS20 shared certain similar sulfation features with the Wnt binding motif.

## Discussion

In the present study, we identified the epitope of a HS-specific human monoclonal antibody (HS20) using a group of synthetic oligosaccharides with different lengths and sulfated modifications. Additionally, we conducted a parallel comparison for Wnt activation, because HS20 is a Wnt-blocking antibody. Although the HS binding motif of HS20 and Wnt3a varied somewhat in modification position and the length of their sulfated domains, they shared certain common features in HS binding. Our analysis provides interesting insights into the inhibition of Wnt binding on HS by the HS20 antibody.

The most common modifications found on HS and CS structures are the 2-O and 6-O-sulfations. Our data clearly showed that HS20 recognized 2-O and 6-O-sulfated HS but not CS. This finding suggested a potentially interesting application of HS20 for distinguishing between HS and CS, especially when studying HSPGs like sydecans that contain both HS and CS chains. HS20 recognized a wide range of HS in length, from 4 to 12 sugar residues, which covers the length of a typical sulfated domain (around 4 to 8 sugars residues). The HS binding site for several factors (e.g., FGF, hepatocyte growth factor or HGF, anti-thrombin) consists of a range between 6 to 12 sugar residues^12^. Our data indicated that the functional length of HS for Wnt binding was also within this range. We found that the HS20 binding site required a shorter motif than the Wnt binding site. It would be interesting to examine whether the HS20 site could potentially overlap the binding site(s) of other growth factors.

Cation binding and the conformation of HS may play important roles in polysaccharide-protein interactions^43^. In the present study, the binding of the HS20 antibody on HS did not seem to be dependent on charge. Firstly, the binding of HS20 did not correlate with the number of charges on the oligosaccharides. Equal inhibition of HS20 binding was found with both long 12mer-5 and 12mer-6 (22 and 23 charges) and with the short oligosaccharides (8mer-5 and 4mer-6) that contain as little as 8 charges (Figs 4 and 5). Secondly, Fig. 1d shows that the binding of HS20 to glypican-3 can be inhibited by HS but not by negatively charged chondroitin sulfate (bikunin). Therefore, we think that the type of sulfations and presence of iduronic acid in HS also play roles in determining the binding to the HS20 antibody. Furthermore, it is possible that the three-dimensional architecture of HS oligosaccharides could play an important role in the binding of HS20 and Wnt. Although the fine structures of HS are different from tissues, they are all biosynthesized using enzymes found in similar biosynthetic pathways. This makes our HS structurally similar to those isolated from natural sources. Future structural analysis for the complex of oligosaccharides with the HS20 antibody or Wnt molecules will be needed to elucidate the binding structure of HS for HS20 or Wnt molecules.

Previous studies have demonstrated the essential role of HS-specific 6-O-endosulfatase for Wnt signaling^27^,^28^. The 6-O-endosulfatase primarily removes the 6-O-sulfo group from the region that contains both 2-O-sulfation and 6-O-sulfation^44^. Therefore, it was interesting for us to find that 6-O-sulfation was required for Wnt activation in the present study. Our results showed that Wnt activation only required 6-O-sulfation, but that HS20 needed both 2-O and 6-O-sulfation for binding. These findings indicated that HS20 may have multiple functions beyond blocking Wnt activation. In fact, it is shown that HS20 inhibits HGF/Met activation and reduces HCC cell migration and motility by targeting the HS chains of GPC3^45^. This observation is consistent with another report showing that N-, 2-O and 6-O sulfation could lead to stronger HGF binding^46^. Additionally, FGF/FGFR binding-HS, which needs both 2-O and 6-O-sulfations^12^,^47^, could possibly be inhibited by HS20 as well. Although 3-O-sulfation was not essential for HS20 binding or Wnt activation, it clearly enhances both activities. Sulfation in the 3-O position is a rare modification in HS^8^ and has been related to many essential biological events including anti-thrombin activity^48^ and viral infections, such as herpes simplex virus^49^,^50^. It should be interesting to investigate if HS20 has any effect on these biological processes.

Several HS-specific antibodies have been reported^51^,^52^,^53^,^54^,^55^,^56^, but few have been characterized. Among them, HS4C3 is characterized for its binding property and activity for disturbing coagulation^54^, and NS4F5 is shown to recognize the (GlcNS6S-IdoA2S)_3_ motif, which is highly expressed in tumor tissues^57^. HS20 recognizes a similar saccharide backbone, (GlcNS6S-IdoA2S)_3–6_, so future studies are necessary to comprehensively evaluate the tissue specificity of the HS20 antibody in clinical samples.

In conclusion, this is the first report of the Wnt binding site on HS. These results reveal the HS oligosaccharide binding motif for Wnt, establish a new methodology using a Wnt-blocking antibody and synthetic HS oligosaccharides, and support a rationale for targeting the Wnt binding site on HS for the treatment of cancer and other diseases.

## Material and Method

### Structurally defined HS oligosaccharides

A total of 14 HS oligosaccharides, ranging from tetrasaccharides (4mers) to dodecasaccharides (12mers), were synthesized for the present study using a chemoenzymatic method^40^,^41^. The purity of each oligosaccharide was determined by high resolution DEAE-HPLC method (Fig. 2). The structures were confirmed by electrospray ionization mass spectrometry (ESI-MS) (Table 1). The schematic cartoons of HS oligosaccharides in the figures show the primary saccharide sequences and do not represent the conformations of oligosaccharides.

### MS analysis

The MS analyses were performed at a Thermo LCQ-Deca. Synthetic LMWH constructs and intermediates were directly diluted in 200 μl of 9:1 MeOH/H_2_O. A syringe pump (Harvard Apparatus) was used to introduce the sample by direct infusion (35 μl/min). Experiments were carried out in negative ionization mode with the electrospray source set to 5 KV and 275 °C. Sulfated oligosaccharide (1 μl) was diluted in a different working solution containing 200 μl of 70% acetonitrile and 10 mM imidazole. Experiments for sulfated oligosaccharides were carried out in negative ionization mode with the electrospray source set to 2 KV and 200 °C. The automatic gain control was set to 1 × 10^7^ for full scan MS. The MS data was acquired and processed using Xcalibur 1.3.

### HPLC analysis

Both DEAE-HPLC and polyamine-based anion exchange (PAMN)-HPLC were used to analyze the purity of the products. For the PAMN-HPLC method, a PAMN column was purchased from Waters (Milford, MA). The column was eluted with a linear gradient of potassium phosphate monobasic. For the DEAE-HPLC method, a DEAE NPR column was purchased from Tosohaas (Stuttgart, Germany). The column was eluted with a linear gradient of sodium chloride in a buffer containing 20 mM sodium acetate pH 4.5. Depending on the size and sulfation levels, different gradient conditions were utilized to achieve the maximum resolution.

### Cell lines and reagents

The CHO-K1 cell line was obtained from ATCC (Manassas, VA). The pgsA-745 cell line was provided by Dr. Jeffrey D. Esko, University of California, San Diego^37^. The HEK293 Supertopflash stable cell line was a gift from Dr. Jeremy Nathans, Johns Hopkins Medical School^42^. L-cell line and L-Wnt3a cell line were provided by Dr. Yingzi Yang, NHGRI, NIH. The cell lines were cultured in DMEM supplemented with 10% fetal bovine serum, 100 U/ml penicillin, 0.1 mg/ml streptomycin, and 2 mmol/L L-glutamine. Bikunin was a gift from Dr. Robert J. Linhardt, Rensselaer Polytechnic Institute^39^. HS was purchased from Sigma (St. Louis, MO).

### Protein production

HS20 and YP7 antibodies were purified in-house as previously described^32^,^58^. GPC3-hFc, GPC3ΔHS-hFc, GPC1-hFc genes were amplified by adding the IL-2 signal peptide sequence at the 5′ end and were inserted into a pVRC8400 expression vector (provided by Dr. Gary J. Nabel, the National Institute of Allergy and Infectious Diseases). The plasmids were transiently transfected into HEK-293T cells. The media was collected and the proteins were purified using a Protein A Hi-Trap column (GE Healthcare, Pittsburgh, PA) according to the manufacturer’s instructions. The quality and quantity of purified proteins were determined by SDS-PAGE and A280 absorbance on a NanoDrop (Thermo Scientific, Asheville, NC), respectively.

### ELISA

Human IgG, HS20, or YP7 (5 µg/ml or indicated concentration, 50 µl/well) was added to 96-well ELISA plates coated with 1 μg/ml of GPC3-hFc, GPC3ΔHS-hFc, a commercial glypican protein (GPC1, GPC3, GPC5 and GPC6; R&D Systems, Minneapolis, MN) or bovine serum albumin (BSA) and incubated at room temperature for 1 hour. The plates were then washed five times with PBST (0.05% Tween-20) followed by incubation at room temperature for 1 hour with 50 μl of goat anti-human kappa chain HRP conjugate (1:5000 dilution, Jackson Laboratory, Bar Harbor, ME) or goat anti-mouse HRP conjugate (1:5000 dilution, Jackson Laboratory, Bar Harbor, ME). After washing three times with PBST, 50 μl/well of 3,3′,5,5′-tetramethylbenzidine detection reagent (KPL, Gaithersburg, MD) was added, and the plate was incubated for 10 minutes at room temperature. Absorbance was read at 450 nm.

### Competition ELISA

Different concentrations of reagents (starting from 100 μg/ml, followed by serial 1:2 dilutions) were incubated with 5 μg/ml of HS20 for 30 minutes at room temperature. The mixture was added to the wells coated with 1 μg/ml of GPC3 or GPC1 and incubated at room temperature for 1 hour. The plates were then washed five times with PBST followed by incubation at room temperature for 1 hour with 50 μl of goat anti-human kappa chain HRP conjugate (1:5000 dilution, Jackson Laboratory, Bar Harbor, ME). The plate was washed and developed following the procedure described above for ELISA.

### Topflash luciferase assay

Wnt3a conditional medium (CM) was collected from the culture supernatant in the presence of 10% FBS. HEK293 Supertopflash cells were seeded into a 48-well plate. When the cells grew to 70% confluency, 50% Wnt3a CM (1:1 dilute with DMEM, which contains 10% FBS) was pre-incubated with 100 μg/ml indicated oligosaccharides or heparan sulfate for 30 minutes and then added to the cells. After 6 hours, cells were lysed and luciferase activity was detected with the Luciferase Reporter Assay Kit (Promega, Madison, WI) according to the manufacturer’s protocol. Data were normalized by the total protein of each well.

### Statistical analysis

All of the representative results were repeated at least three times. All group data (except those indicated) was expressed as the mean ± standard deviation (s.d.) of at least triplicate, and similar results were obtained in at least three independent experiments. All statistical analyses were conducted using GraphPad Prism 6.0. Differences between groups were analyzed using the two-tailed Student t-test of means, with P* < 0.05 defined as significant.

## Additional Information

**How to cite this article**: Gao, W. *et al.* Epitope mapping by a Wnt-blocking antibody: evidence of the Wnt binding domain in heparan sulfate. *Sci. Rep.* **6**, 26245; doi: 10.1038/srep26245 (2016).

## Figures and Tables

**Table 1:** Summary of ESI-MS analysis of synthesized oligosaccharides.

Oligosaccharides	Abbreviated structures	Calculated MW	Measured MW
12mer-1	GlcNAc-GlcA-GlcNAc-GlcA-GlcNAc-GlcA-GlcNAc-GlcA-GlcNAc-GlcA-GlcNAc-GlcA-pNP	2415.0	2415.6 ± 1.0
12mer-2	GlcNS-GlcA-GlcNS-GlcA-GlcNS-GlcA-GlcNS-GlcA-GlcNS-GlcA-GlcNS-GlcA-pNP	2643.1	2642.8 ± 0.7
12mer-3	GlcNS6S-GlcA-GlcNS6S-GlcA-GlcNS6S-GlcA-GlcNS6S-GlcA-GlcNS6S-GlcA-GlcNS6S-GlcA-pNP	3123.5	3122.8 ± 0.2
12mer-4	GlcNS-GlcA-GlcNS-IdoA2S-GlcNS-IdoA2S-GlcNS-IdoA2S-GlcNS-IdoA2S-GlcNS-GlcA-pNP	2963.4	2963.3 ± 0.7
12mer-5	GlcNS6S-GlcA-GlcNS6S-IdoA2S-GlcNS6S-IdoA2S-GlcNS6S-IdoA2S-GlcNS6S-IdoA2S-GlcNS6S-GlcA-pNP	3443.7	3442.9 ± 0.4
12mer-6	GlcNS6S-GlcA-GlcNS3S6S-IdoA2S-GlcNS6S-IdoA2S-GlcNS6S-IdoA2S-GlcNS6S-IdoA2S-GlcNS6S-GlcA-pNP	3523.8	3524.7 ± 2.2
12mer-7	GlcNS6S-GlcA-GlcNS3S6S-IdoA2S-GlcNS6S-IdoA2S-GlcNS3S6S-IdoA2S-GlcNS6S-IdoA2S-GlcNS6S-GlcA-pNP	3603.8	3603.1 ± 1.5
10mer-5	GlcNS6S-GlcA-GlcNS6S-IdoA2S-GlcNS6S-IdoA2S-GlcNS6S-IdoA2S-GlcNS6S-GlcA-pNP	2866.2	2866.2 ± 0.9
8mer-5	GlcNS6S-GlcA-GlcNS6S-IdoA2S-GlcNS6S-IdoA2S-GlcNS6S-GlcA-pNP	2288.8	2289.3 ± 0.4
6mer-5	GlcNS6S-GlcA-GlcNS6S-IdoA2S-GlcNS6S-GlcA-pNP	1711.4	1710.9 ± 0.5
4mer-5	GlcNS6S-IdoA2S-GlcNS6S-GlcA-pNP	1214.0	1214.0 ± 0.6
4mer-6	GlcNS3S6S-IdoA2S-GlcNS6S-GlcA-pNP	1294.0	1293.8 ± 0.3
